# Progress in Medicine

**Published:** 1900-08-04

**Authors:** 


### Progress in Medicine.

### NEUROLOGY.

**Degeneration of the Neurone.**—In his Croonian lectures on the degeneration of the neurone, Mott[1] states that the term neurone was first introduced by Waldeyer for the nerve cell and all its processes, including the protoplasmic processes or dendrons, and the single axis-cylinder process or axon, with its cone of origin, its collaterals or side branches, and its terminal arborisation. The theory of the neurone is that the nervous system consists of innumerable such anatomically independent nervous units in contiguity, but not in continuity. The investigations of Fleschig showed that there is a correlation between the functions of communities of neurones and the myelination of their axis-cylinder processes; bundles of fibres of different physiological functions are myelinated at different periods of time. The myelin sheath appears to be necessary for the functional activity of nerve tracts, for its development proceeds *pari passu* with the development of function. When the myelination of a nervous system is complete further information by a study of the condition of the myelin sheath can only be obtained by degeneration produced either by disease or experimentally.

The researches of Waller formed the commencement of our knowledge of the degeneration of the neurone. He stated that every part of a nerve separated from its cell of origin undergoes degeneration, whilst the rest remains normal. This statement requires modification, for Dickinson long ago showed that long disuse of a nerve, as after amputation of a limb, is sufficient to lead to its atrophy, though those nervous structures which immediately regulate its nutrition are complete. And Gudden showed that ablation of a nerve in a young animal led to atrophy of its cells of origin. Though axis-cylinder processes may regenerate, yet there is no evidence to show that regeneration of nerve cells ever takes place. Each nerve cell is endowed with a specific durability. The neurones of any particular system may die prematurely, owing to an inherited or acquired want of durability. This regressive process of decay first affects those parts most remote from the trophic and genetic centre of the cell.

Only since the introduction of the Nissl method of staining has it become known that changes occur in the ganglion cells almost immediately after section of the nerve. The changes differ according as the axis cylinder process regenerates or not. If it does, there is first a "reaction phase," followed by a "reparation phase," whilst if regeneration be impossible instead of a reparation phase degeneration of the nerve cell takes place. The application of this method has proved of value in determining the cells of origin of tracts of fibres; for instance, that the descending antero-lateral tract of the spinal cord arises from Deiter's nucleus. It is then evident that the whole cell suffers when its axis-cylinder process is destroyed, though actual degeneration only occurs in that part of the axis-cylinder process which has been severed from its cell of origin.

The histological elements which make up the nervous system may be divided into two groups: the nervous units, or neurones; and the supporting, protecting, and nutrient tissues. Organic disease may start in a primary degeneration of the neurones, or these may be affected secondarily by diseases which, starting in the supporting, protecting, and nutrient tissues, cause their degeneration by direct injury, inflammatory compression, or by cutting off the blood supply.

The primary degenerative changes occurring in the neurone are usually the result of poisonous conditions of the blood and lymph. Such poisons may be produced within the body either by perverted functions of the organs, or by the actions of micro-organisms on the tissues, or be introduced from without. Most poisons have a selective influence, that is, attack first of all some particular group of neurones. For instance, the poison of tetanus selects the motor nucleus of the fifth nerve. The corollary may be drawn that the protoplasm of every nerve structure having different functions varies in slight degree.

In his third Croonian lecture Mott discussed the chemistry of nerve degeneration. He has found that choline is a product of nerve degeneration, the result of breaking up of protagon or lecithin, that it escapes into the cerebro spinal fluid, thence into the blood in larger quantities than it can be disposed of by oxidation, and that its accumulation in the blood might produce auto-intoxication. The experimental injection of choline into the circulation was a fall of arterial blood pressure, partly due to its action on the heart but mainly due to dilatation of peripheral vessels, especially in the intestinal area.

In the cerebro-spinal fluid in cases of general paralysis, not only is choline found, but also a relatively large amount of proteid. This excess of proteid matter is due to the presence of a nucleo-proteid not found in the normal cerebro-spinal fluid. The nucleo-proteid probably favours congestive stasis and even coagulation in the veins of the brain in general paralysis. Thus the disintegrating nervous tissue may be the means of setting up a vicious circle, whereby, owing to the circulatory disturbances, acute destructive processes of the nervous tissue are brought about. This hypothesis is supported by the fact that most of the nerve-cells in the degenerating tissue of the cortex are devoid of the normal Nissl granules, which have been shown by Held to be the result of coagulated nucleo-albumen.

The Marchi reaction applied to the central and peripheral nervous systems shows rather different results; one striking difference is the comparatively rapid disappearance of the products of degeneration in peripheral nerves as compared with the central nervous system. Mott suggests that this difference may probably be correlated with the presence of a nucleated sheath of Schwann to each nerve-fibre in the peripheral nerves, and its absence in the central nervous system.

[1] Mott, Lancet, June 23 and 30, and July 7, 1900.

